# Supplementary material for: Characterization of 475 Novel, Putative Small RNAs (sRNAs) in Carbon-Starved Salmonella enterica Serovar Typhimurium
Source: Antibiotics (Basel). 2021 Mar 16;10(3):305. doi: 10.3390/antibiotics10030305 (PMC8000849; doi:10.3390/antibiotics10030305)
Supplement: Supplementary file 1 [file antibiotics-10-00305-s001.zip › antibiotics-1128165-supplementary/antibiotics-supp legends (gb).docx]

**Supplemental Figure 1. Workflow for determining candidate sRNAs**.

**Supplemental Figure 2**. **Heat maps depicting gene expressions of sRNAs dynamically expressed during CSR**. (**a**) Heatmap of intragenic sRNAs. (**b**) Heatmap of intergenic sRNAs. (**c**) Heatmap of previously annotated sRNAs. Log, log phase; 5-hr, 5 hour C-starved; 24-hr, 24 hour C-starved. Individual sRNA gene names are included along the Y-axis.

**Supplemental Figure 3**. **qRT-PCR verification of expressions of sRNAs identified as being dynamically expressed during the CSR in SL1344**. Results are shown as normalized relative expression compared to levels of the reference gene rpoD. Log_phase = non-starved cells harvested on log phase and high C medium; 5h stv = cells starved for 5h in low C medium; 24h stv = cells starved for 24h in low C medium.

**Supplemental Figure 4**. **qRT-PCR verification of sRNA expression vectors**. qRT-PCR of specified sRNAs after IPTG induction of corresponding pHMB1 expression constructs.

**Supplemental Figure 5. sRNA1186573 participates in biofilm production.** (**a**) Congo Red test for Curli production. Results of deletion mutants for sRNA924744, sRNA1170414, sRNA1186573, sRNA176086, sRNA2594511, sRNA3551252, sRNA4130247, and sRNA4720054 are shown. (**b**) Ratio of OD570 measurements (sRNA overexpression/deletion).

**Supplemental Table 1**. **Comprehensive catalog of 871 unique *Salmonella* enterica SL1344 sRNAs**. Initially reported name (ID), chromosomal (Start) and (Stop) positions, (Source) of initial annotation, and positive strand (Sequence) for 396 previously reported and 475 novel sRNA loci described in this report are detailed. In the event of a sRNA locus having been reported in more than one study and having received multiple designations as a result, the ID corresponding to the initial description was selected. Ryan et al. [39]; Amin et al., [11]; Barnhill et al., [15]; Houserova et al., this report; Kroger et al., [17].

**Supplemental Table 2. Novel intergenic sRNAs in Carbon-starved *Salmonella* enterica**. sRNA (name based on chromosomal position), sequence, expression in RPM (Log, 5h, 24h), and putative gene targets are indicated.

**Supplemental Table 3. Novel intragenic sRNAs in Carbon-starved *Salmonella* enterica**. sRNA (name based on chromosomal position), sequence, expression in RPM (Log, 5h, 24h), and putative gene targets are indicated.
