# Supplementary material for: Characterization of 475 Novel, Putative Small RNAs (sRNAs) in Carbon-Starved Salmonella enterica Serovar Typhimurium
Source: Antibiotics (Basel). 2021 Mar 16;10(3):305. doi: 10.3390/antibiotics10030305 (PMC8000849; doi:10.3390/antibiotics10030305)
Supplement: Supplementary file 1 [file antibiotics-10-00305-s001.zip › antibiotics-1128165-supplementary/Supplementary Table 5.docx]

**Supplemental Table 5**. qPCR Primers.

| **Gene** | **Sequence (5’-3’)** |
| --- | --- |
| rpoD | F: GTATGCCCTCGCTTTAAATGTC |
|  | R: TCAGATAGCCTTGCTCCTTACC |
| STnc3920 | F: TAAAGCTCCCCGTAATTTAGCA |
|  | R: CAGGGCGTTAATTACCTTTGAA |
| STnc1460 | F: TGTTCCCTTCGCAGGTATTAAC |
|  | R: ATGAAGTGGAAAAGGCTGAGAC |
| STnc700 | F: TAAACACCACCATCATCACCAT |
|  | R: GATCTGAATGTCTTCCAGCACA |
| IsrL | F: CCGTTAACTGGCATCCTTCTAT |
|  | R: GGCGACCTCTATTTGTTCATTC |
| sRNA294324 | F: GTTTTGGCGGATTGAGAAGAC |
|  | R: CCTTGTCTCAGCGTAAAATCCT |
| sRNA3981754 | F: CAGCTACTGATTGAAAGTTATACCAAAG |
|  | R: GTTATTTATGCGCGTTGAGAATCC |
| sRNA3417670 | F: GATGTGATTGCCAAACAGACTC |
|  | R: GCCATAATTAACGACGCGTC |
| sRNA1320654 | F: CCGTTCATATGAATAGTAGAGGCTC |
|  | R: GCAGAGACGGCGTTCAGT |
| sRNA3417448 | F: CCAGAGACTACTAAATTAACGCC |
|  | R: CTTCTTCAAGCGTATCGGTCA |
| sRNA294677 | F: AGAGAGTAGGACAAAAGCGAAAG |
|  | R: TGGCAGTTTATGAATCACTTCGTG |
