# Supplementary figures and images for: Characterization of 475 Novel, Putative Small RNAs (sRNAs) in Carbon-Starved Salmonella enterica Serovar Typhimurium
Source: Antibiotics (Basel). 2021 Mar 16;10(3):305. doi: 10.3390/antibiotics10030305 (PMC8000849; doi:10.3390/antibiotics10030305)

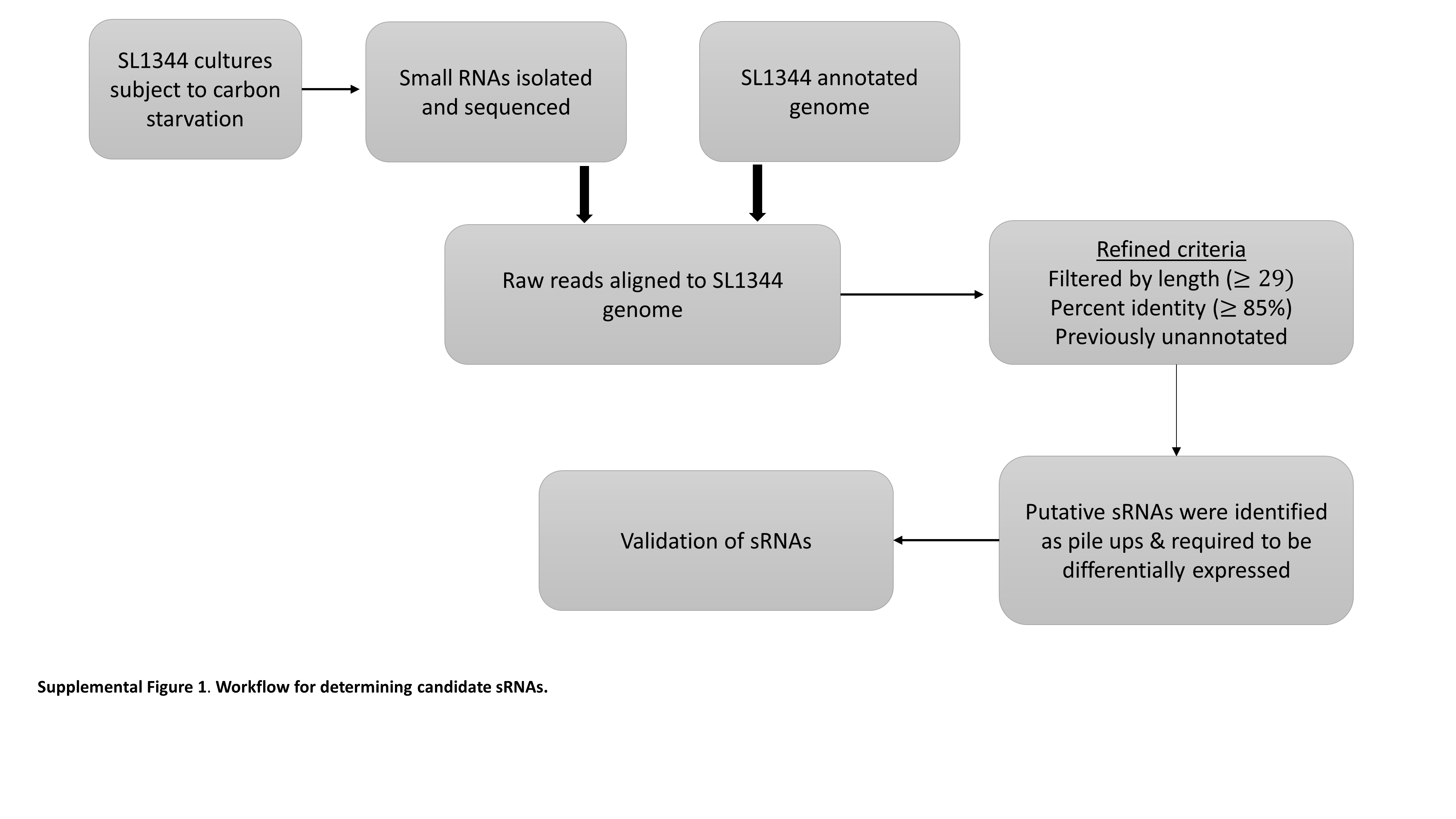

Supplement: Supplementary file 1 [file antibiotics-10-00305-s001.zip › antibiotics-1128165-supplementary/Supplementary Figure 1.TIF]

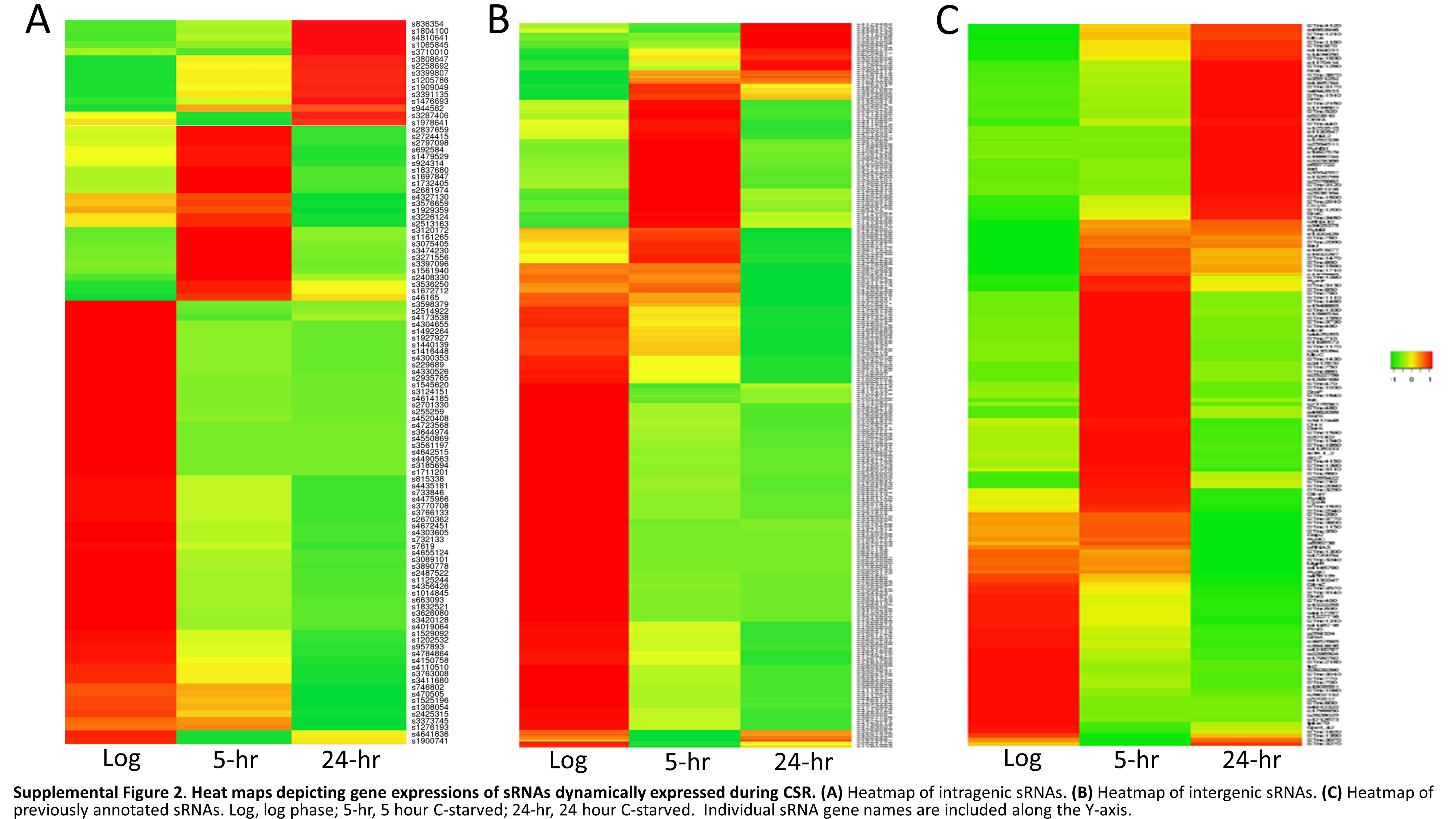

Supplement: Supplementary file 1 [file antibiotics-10-00305-s001.zip › antibiotics-1128165-supplementary/Supplementary Figure 2.TIF]

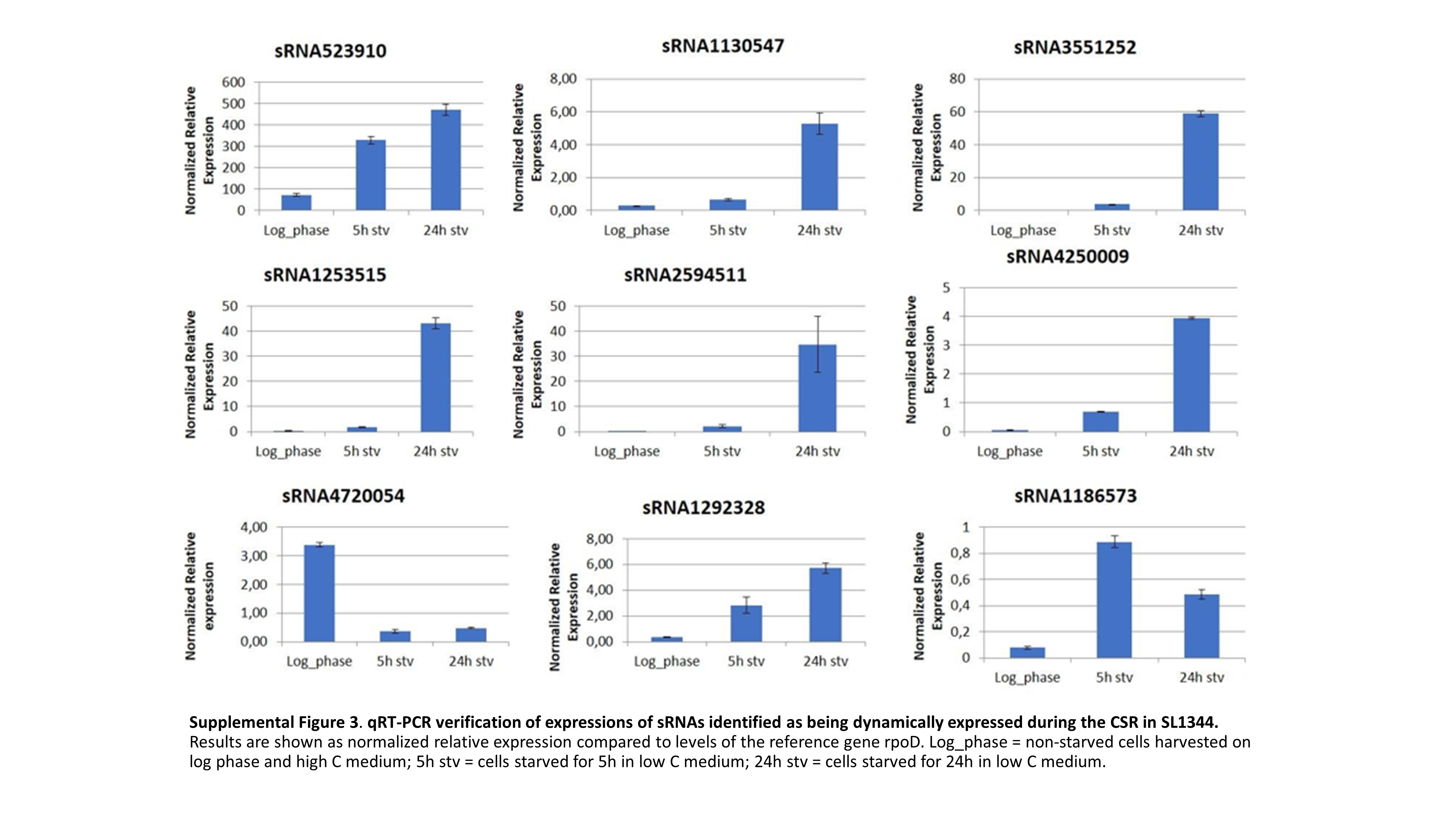

Supplement: Supplementary file 1 [file antibiotics-10-00305-s001.zip › antibiotics-1128165-supplementary/Supplementary Figure 3.TIF]

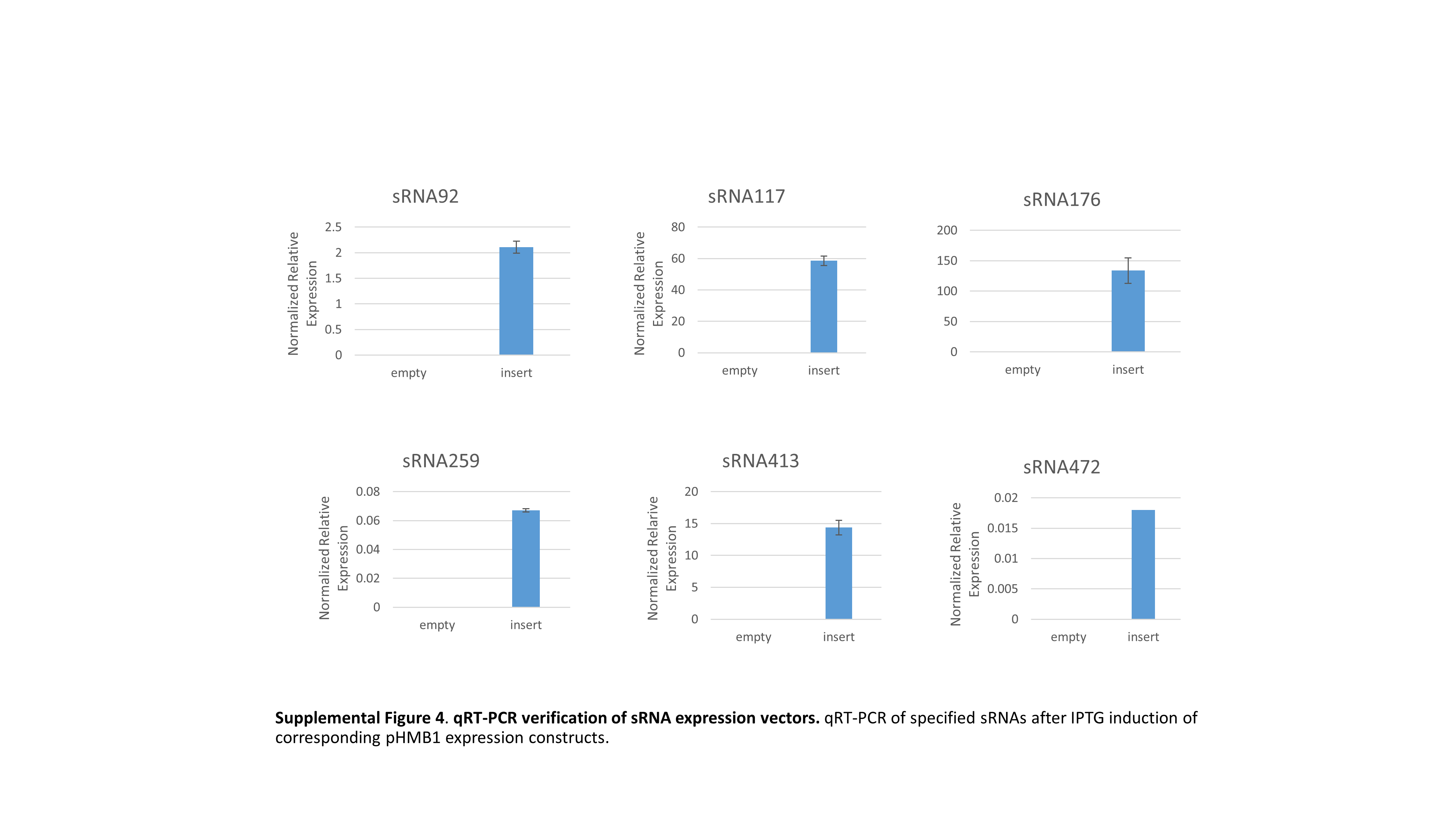

Supplement: Supplementary file 1 [file antibiotics-10-00305-s001.zip › antibiotics-1128165-supplementary/Supplementary Figure 4.TIF]

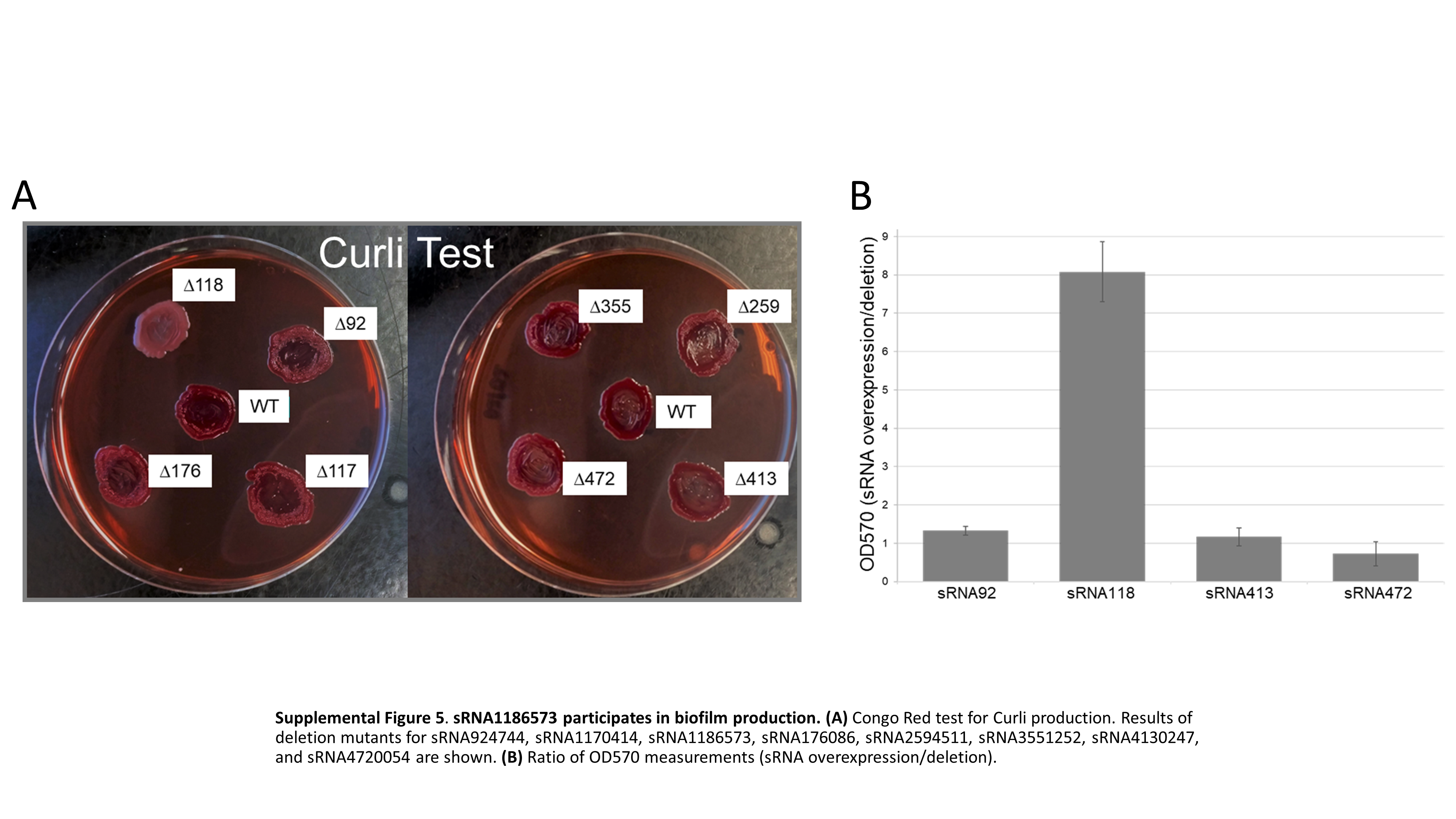

Supplement: Supplementary file 1 [file antibiotics-10-00305-s001.zip › antibiotics-1128165-supplementary/Supplementary Figure 5.TIF]
